# Association of Pack-Years of Cigarette Smoking With Survival and Tumor Progression Among Patients Treated With Chemoradiation for Head and Neck Cancer

**DOI:** 10.1001/jamanetworkopen.2022.45818

**Published:** 2022-12-08

**Authors:** Sung Jun Ma, Han Yu, Brian Yu, Olivia Waldman, Michael Khan, Udit Chatterjee, Sharon Santhosh, Jasmin Gill, Austin J. Iovoli, Mark Farrugia, Alina Shevorykin, Ellen Carl, Kimberly Wooten, Vishal Gupta, Ryan McSpadden, Moni A. Kuriakose, Michael R. Markiewicz, Ayham Al-Afif, Wesley L. Hicks, Mary E. Platek, Mukund Seshadri, Christine Sheffer, Graham W. Warren, Anurag K. Singh

**Affiliations:** 1 Department of Radiation Medicine, Roswell Park Comprehensive Cancer Center, Buffalo, New York; 2 Department of Biostatistics and Bioinformatics, Roswell Park Comprehensive Cancer Center, Buffalo, New York; 3 Jacobs School of Medicine and Biomedical Sciences, University at Buffalo, The State University of New York, Buffalo; 4 University at Buffalo, The State University of New York, Buffalo; 5 Department of Health Behavior, Roswell Park Comprehensive Cancer Center, Buffalo, New York; 6 Department of Head and Neck Surgery, Roswell Park Comprehensive Cancer Center, Buffalo, New York; 7 Department of Oral and Maxillofacial Surgery, School of Dental Medicine, University at Buffalo, The State University of New York, Buffalo; 8 Department of Neurosurgery, Jacobs School of Medicine and Biomedical Sciences, University at Buffalo, The State University of New York, Buffalo; 9 Department of Nutrition and Dietetics, D’Youville University, Buffalo, New York; 10 Department of Oral Oncology, Roswell Park Comprehensive Cancer Center, Buffalo, New York; 11 Hollings Cancer Center, Department of Radiation Oncology, Medical University of South Carolina, Charleston

## Abstract

**Importance:**

After 10 pack-years of smoking was initially established as a threshold for risk stratification, subsequent clinical trials incorporated it to identify candidates for treatment deintensification. However, several recent studies were unable to validate this threshold externally, and the threshold for smoking exposure remains unclear.

**Objective:**

To estimate the threshold of pack-years of smoking associated with survival and tumor recurrence among patients with head and neck cancer.

**Design, Setting, and Participants:**

This single-institution, cohort study included patients with nonmetastatic head and neck cancer receiving chemoradiation from January 2005 to April 2021. Data were analyzed from January to April 2022.

**Exposures:**

Heavy vs light smoking using 22 pack-years as a threshold based on maximizing log-rank test statistic.

**Main Outcomes and Measures:**

Overall survival (OS), progression-free survival (PFS), locoregional failure (LRF), and distant failure (DF).

**Results:**

A total of 518 patients (427 male [82.4%]; median [IQR] age, 61 [55-66] years) were included. Median (IQR) follow-up was 44.1 (22.3-72.8) months. A nonlinear Cox regression model using restricted cubic splines showed continuous worsening of OS and PFS outcomes as pack-years of smoking increased. The threshold of pack-years to estimate OS and PFS was 22. Cox multivariable analysis (MVA) showed that more than 22 pack-years was associated with worse OS (adjusted hazard ratio [aHR] 1.57; 95% CI, 1.11-2.22; *P* = .01) and PFS (aHR, 1.38; 95% CI, 1.00-1.89; *P* = .048). On Fine-Gray MVA, heavy smokers were associated with DF (aHR, 1.71; 95% CI, 1.02-2.88; *P* = .04), but not LRF (aHR, 1.07; 95% CI, 0.61-1.87; *P* = .82). When 10 pack-years of smoking were used as a threshold, there was no association for OS (aHR, 1.23; 95% CI, 0.83-1.81; *P* = .30), PFS (aHR, 1.11; 95% CI, 0.78-1.57; *P* = .56), LRF (aHR, 1.19; 95% CI, 0.64-2.21; *P* = .58), and DF (aHR, 1.45; 95% CI, 0.82-2.56; *P* = .20). Current smoking was associated with worse OS and PFS only among human papillomavirus (HPV)-positive tumors (OS: aHR, 2.81; 95% CI, 1.26-6.29; *P* = .01; PFS: aHR, 2.51; 95% CI, 1.22-5.14; *P* = .01).

**Conclusions and Relevance:**

In this cohort study of patients treated with definitive chemoradiation, 22 pack-years of smoking was associated with survival and distant metastasis outcomes. Current smoking status was associated with adverse outcomes only among patients with HPV-associated head and neck cancer.

## Key Points

**Question:**

Is there a threshold of pack-years of smoking associated with survival and tumor recurrence among patients with head and neck cancer?

**Findings:**

In this cohort study of 518 participants, 22 pack-years was estimated to be the threshold for estimating cancer treatment outcomes. Consumption greater than 22 pack-years was associated with decreased longevity, reduced progression-free survival, and increased distant metastasis, but not locoregional recurrence.

**Meaning:**

Heavy smoking was associated with survival outcomes and distant metastasis, and further studies are warranted to tailor smoking cessation interventions among all smokers.

## Teaser

This cohort study evaluates the association of pack-years of cigarette smoking with survival and tumor progression among patients treated with chemoradiation for head and neck cancer.

## Introduction

Tobacco smoking has been shown to reduce the efficacy of radiation therapy among patients with head and neck cancer.^1,2,3,4^ Given the importance of smoking, a secondary analysis of the Radiation Therapy Oncology Group 0129 trial established 10 pack-years of smoking as a threshold of risk stratification for survival in the context of human papillomavirus (HPV).^5^ The 10 pack-year threshold has been incorporated to identify patients potentially eligible for treatment deintensification.^6^

However, the authors of the Radiation Therapy Oncology Group 0129 trial suggested that the 10 pack-year threshold should be validated further before its adoption for risk stratification.^5^ In addition, several recent studies have been unable to validate the role of 10 pack-years as an important threshold.^7,8^ When 10 pack-years of smoking was initially established as a threshold, the analyses did not incorporate other relevant oncologic outcomes, such as progression-free survival (PFS), locoregional failure (LRF), and distant failure (DF).^5^ A more easily quantified, verified, and potentially modifiable variable, current smoking status portends worse survival.^4,7,9,10^ To evaluate the threshold of pack-years of smoking and revisit 10 pack-years of smoking and current smoking status as variables, we performed a single-institution, observational cohort study involving patients with head and neck cancer who underwent chemoradiation.

## Methods

This study was approved by the Roswell Park Comprehensive Cancer Center Institutional Review Board. Informed consent was waived since our study met the criteria for minimal risk to the study participants in accordance with 45 CFR §46. This study follows the Strengthening the Reporting of Observational Studies in Epidemiology (STROBE) reporting guideline.

The cohort database was established including all patients with primary head and neck cancer at the Roswell Park Comprehensive Cancer Center between January 2005 to April 2021. Last follow-up was performed in June 2021. Patients were included for analysis if they had a diagnosis of nonmetastatic head and neck cancer treated with curative-intent definitive chemoradiation using intensity modulated radiation therapy^11^ with 70 Gy to gross disease and 56 Gy to elective neck lymph nodes in 35 fractions. Patients were excluded from the analysis if smoking history data were missing or if they underwent surgery or palliative-intent treatments.

Smoking status and history was self-reported as part of routine care and were extracted through retrospective medical record review of initial consultation notes. The following questions were asked to determine the smoking status: “Have you smoked cigarettes in the past?”; “Are you currently smoking cigarettes?”; “How many packs per day do you smoke?”; “How many years have you smoked?” All patients were seen by a single radiation oncologist (A.K.S.) whose policy was to give current smokers several weeks to quit smoking while using cessation services as previously described.^12^ Those who quit were given 30 days before initiation of radiation. Those who could not quit were excluded for analysis, since they were given induction chemotherapy if feasible and appropriate.

Other variables of interest included age, sex, race, Karnofsky Performance Status, number of comorbidities, primary cancer site, cancer staging according to the American Joint Committee on Cancer 7th edition, HPV status, and chemotherapy agent. The multivariable analysis (MVA) models included all of the aforementioned variables. All missing values were coded as unknown for analysis. Race was self-identified as African American, American Indian/Alaska Native, Asian, Hispanic, unknown or declined to answer, and White. Because of the small subgroup sample sizes, African American, American Indian/Alaska Native, Asian, and Hispanic patients were grouped together as a single category. Race was assessed in this study because there may be racial differences in clinical outcomes among patients with head and neck cancer.

The primary outcomes included overall survival (OS) and progression-free survival (PFS). OS is defined as the time intervals from diagnosis to death from any cause. PFS is defined as the time to the last follow-up and tumor progression or death. Secondary outcomes included LRF and DF. LRF and DF were defined as time intervals from diagnosis to progression within and outside head and neck regions, respectively. Tumor progression was evaluated in a multidisciplinary setting, including discussion based on radiographic findings and biopsy results of metastatic sites if available.

### Statistical Analysis

Cox univariable analysis, Kaplan-Meier method, and log-rank tests were performed to evaluate the association among HPV, smoking status at diagnosis, and 10 pack-years of smoking with OS and PFS. Reference groups were HPV-positive with either less than 10 pack-years of smoking or never/former smoking at diagnosis compared with other subgroups. Holm-Bonferroni correction was used for multiple comparisons.

To visualize the association between survival outcomes and pack-years of smoking as a continuous variable, a nonlinear Cox regression model using restricted cubic splines was performed.^13,14,15^ Restricted cubic splines is a smooth, piecewise polynomial function evaluating the association between a variable and an outcome without any prior assumption in the association.^16,17^ The model was constructed using 3 knots at the 10th, 50th, and 90th percentiles according to the lowest Akaike information criterion.^16,18^

A threshold for pack-years of smoking was estimated by using an outcome-oriented approach by maximizing the log-rank test statistic and the survival differences,^19^ as previously used for finding thresholds on neutrophil-lymphocyte ratio^20^ and metabolic tumor volume^21^ for head and neck cancer. Such thresholds were evaluated for both OS and PFS separately, and patients were then stratified into 2 cohorts, heavier vs never/lighter smokers at diagnosis, by above vs below the threshold for their pack-years of smoking, respectively. Comparison of baseline characteristics were performed using Fisher exact test and Mann-Whitney *U* test as appropriate. OS and PFS were evaluated using Kaplan-Meier method and log-rank tests. Cox MVA was performed to identify variables associated with OS and PFS. Fine-Gray competing risk MVA was performed to evaluate LRF and DF with death as a competing event. Cox and Fine-Gray MVA were repeated using 10 pack-years of smoking as a threshold.^5^ Among those with available HPV data, subgroup analysis using Cox MVA was performed.

To reduce selection bias, propensity score matching was performed on the basis of all baseline characteristics listed previously. Matching was based on nearest neighbor method in a 1:1 ratio without replacement using a caliper distance of 0.15.^22^ Cox and Fine-Gray regression models were performed to evaluate OS, PFS, LRF, and DF after matching.

All statistical tests were 2-sided and *P* < .05 was considered significant. All analyses were performed using R statistical software version 4.1.2 (R Project for Statistical Computing). Data were analyzed from January to April 2022.

## Results

Of 857 patients who underwent curative-intent radiation therapy in the database, 158 patients underwent surgery, 91 patients underwent radiation therapy alone, and 78 patients underwent induction chemotherapy. Of 530 patients who underwent definitive chemoradiation, an additional 12 patients were excluded due to missing information on pack-years of smoking. Of all patients, a total of 54 (10.4%) were lost to follow-up. A total of 518 patients (427 male [82.4%]; median [IQR] age, 61 [55-66] years) met our criteria (Table). The majority (288 [55.6%]) of patients had oropharyngeal cancer treated with concurrent cisplatin (434 [83.8%]). Median (IQR) follow-up was 44.1 (22.3-72.8) months. Among current (97 [18.7%]) and former (287 [55.4%]) smokers at diagnosis, median (IQR) pack-years of smoking were 35 (20-50) pack-years. The study had small sample sizes of nonsmokers with HPV-negative tumors (9 out of 98 patients, 9.2%) and fairly small sample sizes of 134 never smokers (25.9%) and 97 current smokers (18.7%).

**Table.** Baseline Patient Characteristics

Variables	Before matching			After matching		
	Patients, No. (%)		*P* value	Patients, No. (%)		*P* value
	<22 PY	≥22 PY		<22 PY	≥22 PY	
Sex						
Male	217 (85.1)	210 (79.8)	.13	127 (85.8)	123 (83.1)	.63
Female	38 (14.9)	53 (20.2)		21 (14.2)	25 (16.9)	
Smoker						
Never or former	240 (94.1)	181 (68.8)	<.001	133 (89.9)	131 (88.5)	.85
Current	15 (5.9)	82 (31.2)		15 (10.1)	17 (11.5)	
Age, median (IQR), y	59.8 (54.0-65.2)	62 (55.8-67.1)	.02	62.6 (54.8-68.5)	61.7 (56.1-67.2)	.90
Karnofsky Performance Status						
<90	50 (19.6)	91 (34.6)	<.001	40 (27.0)	40 (27.0)	.99
90-100	202 (79.2)	171 (65.0)		108 (73.0)	107 (72.3)	
Not available	3 (1.2)	1 (0.4)		0	1 (0.7)	
Race^a^						
White	219 (85.9)	230 (87.5)	.61	128 (86.5)	125 (84.5)	.74
Other	36 (14.1)	33 (12.5)		20 (13.5)	23 (15.5)	
Comorbidity						
0	44 (17.3)	38 (14.4)	.52	18 (12.2)	20 (13.5)	.79
1-3	152 (59.6)	155 (58.9)		91 (61.5)	94 (63.5)	
≥4	59 (23.1)	70 (26.6)		39 (26.4)	34 (23.0)	
Site						
Oropharynx	168 (65.9)	120 (45.6)	<.001	87 (58.8)	85 (57.4)	.97
Larynx	27 (10.6)	90 (34.2)		25 (16.9)	26 (17.6)	
Other	60 (23.5)	53 (20.2)		36 (24.3)	37 (25.0)	
T staging						
1-2	159 (62.4)	110 (41.8)	<.001	82 (55.4)	74 (50.0)	.42
3-4	96 (37.6)	153 (58.2)		66 (44.6)	74 (50.0)	
N staging						
0-1	53 (20.8)	98 (37.3)	<.001	37 (25.0)	44 (29.7)	.43
2-3	202 (79.2)	165 (62.7)		111 (75.0)	104 (70.3)	
Human papillomavirus						
Negative	25 (9.8)	73 (27.8)	<.001	24 (16.2)	23 (15.5)	.95
Positive	166 (65.1)	86 (32.7)		76 (51.4)	74 (50.0)	
Not available	64 (25.1)	104 (39.5)		48 (32.4)	51 (34.5)	
Chemotherapy						
Cisplatin	220 (86.3)	214 (81.4)	.15	118 (79.7)	119 (80.4)	.99
Other	35 (13.7)	49 (18.6)		30 (20.3)	29 (19.6)	

Abbreviation: PY, pack-years of smoking.

^a^ Races are self-identified as African American, American Indian/Alaska Native, Asian, Hispanic, unknown or declined to answer, and White. Because of the small subgroup sample sizes, African American, American Indian/Alaska Native, Asian, Hispanic, and unknown or declined to answer were grouped together as a single category.

**Figure 1.**
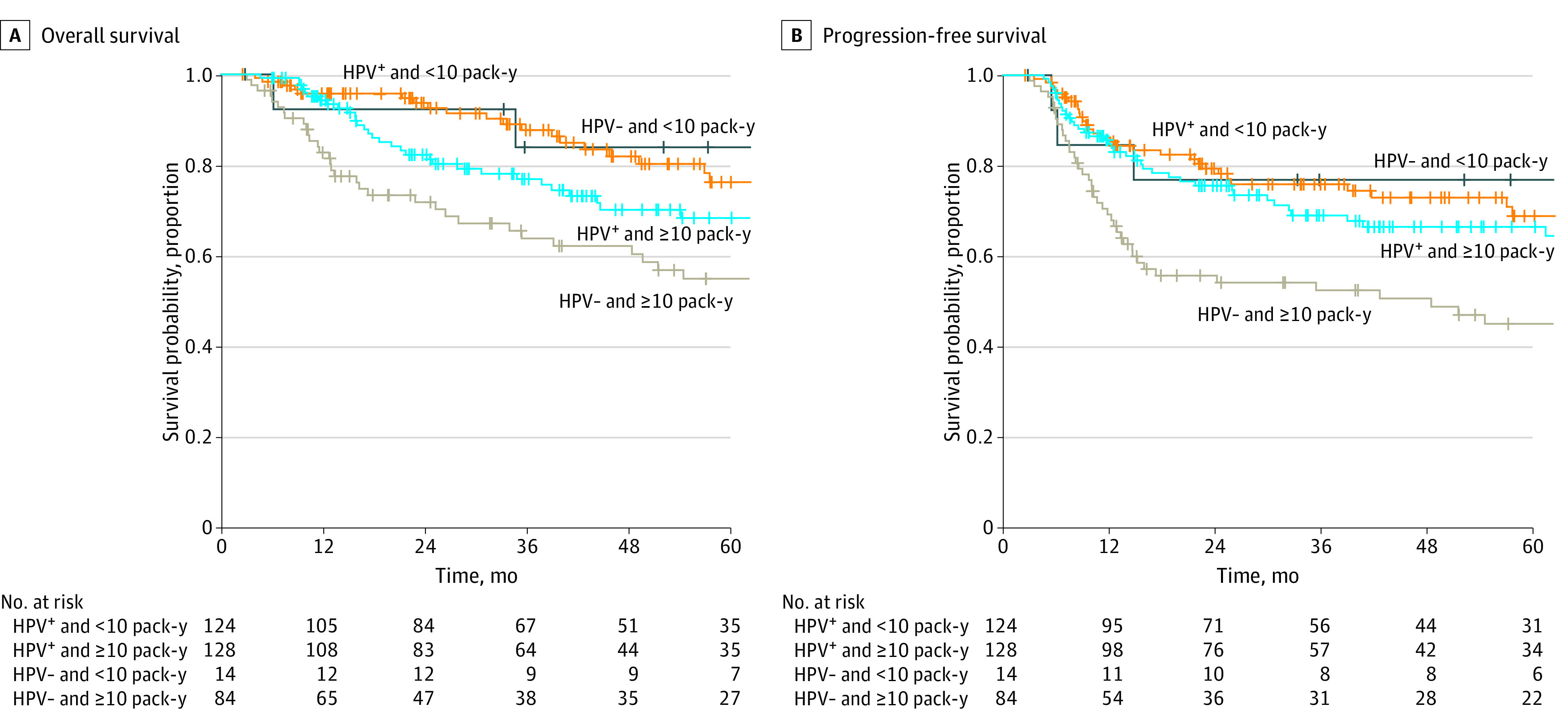
Kaplan-Meier Curves for Survival Outcomes According to Human Papillomavirus (HPV) Status and 10 Pack-Years of Smoking as a Threshold

**Figure 2.**
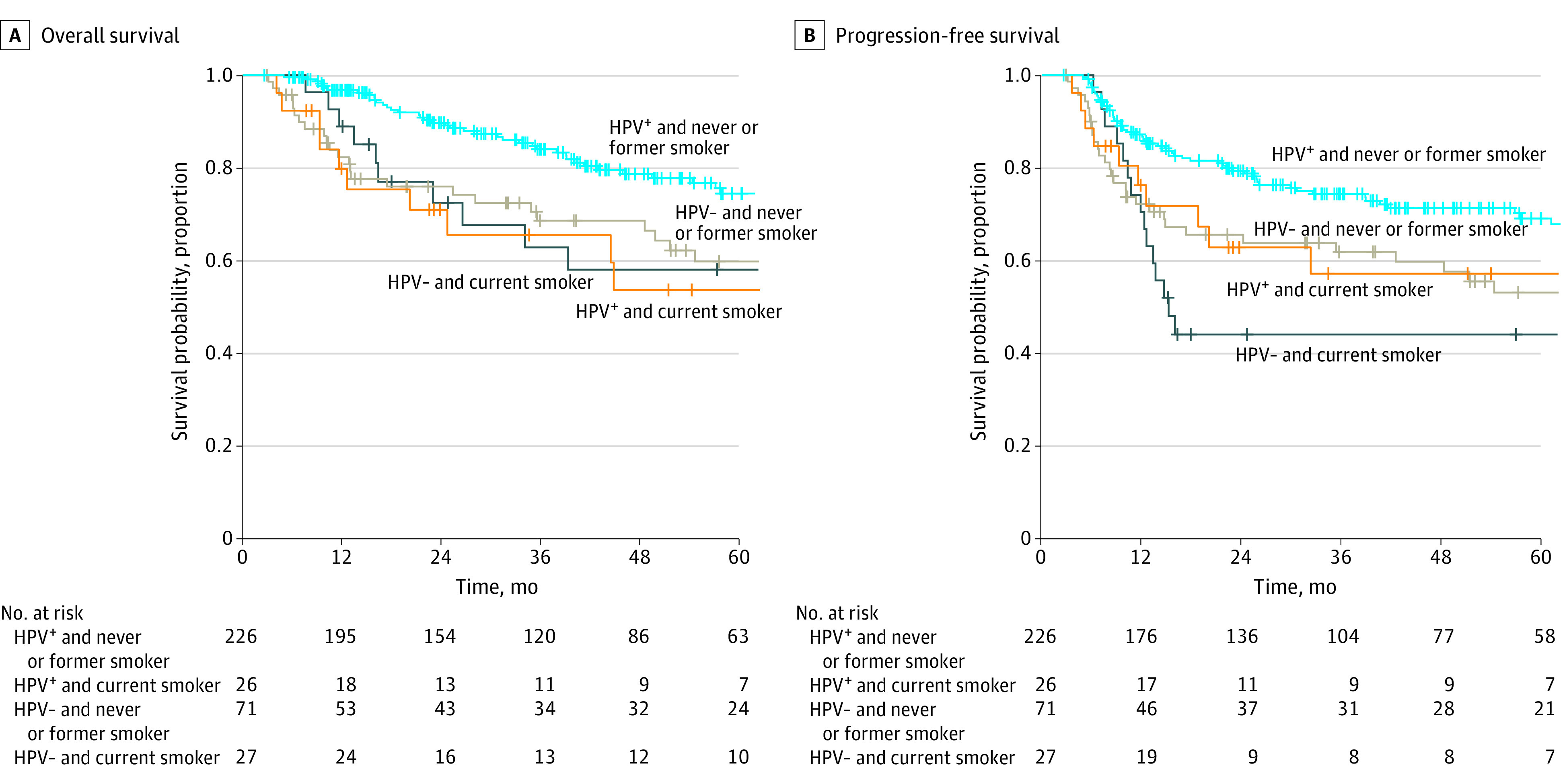
Kaplan-Meier Curves for Survival Outcomes According to Human Papillomavirus (HPV) Status and Smoking Status at Diagnosis

Using Cox univariable analysis (eTable 1 in the Supplement), when compared with patients who were HPV-positive and who had less than 10 pack-years of smoking, only those who were HPV-negative with 10 or more pack-years of smoking were associated with worse OS and PFS. When compared with patients who were HPV-positive and had never or former smoking status, those with HPV-positive and current smoking status had worse OS, but not PFS. Patients with HPV-negative tumors had worse OS and PFS regardless of smoking status. Kaplan-Meier plots were shown in Figure 1 and Figure 2.

**Figure 3.**
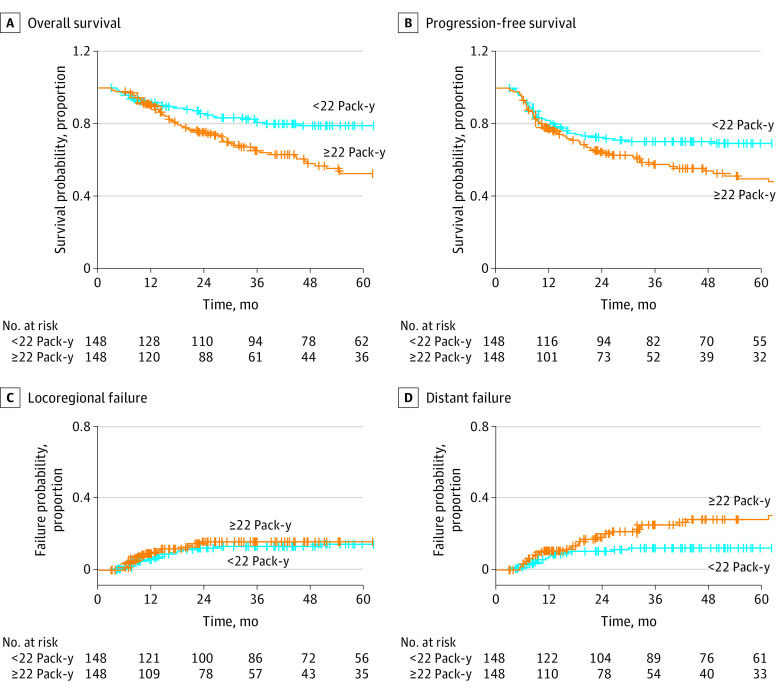
Kaplan-Meier and Cumulative Incidence Curves for Survival and Tumor Progression Outcomes

The nonlinear Cox regression model using restricted cubic splines showed worsening OS and PFS without plateau in a continuous fashion as the pack-years of smoking increased, crossing hazard ratio (HR) of 1.0 at approximately 20 pack-years of smoking (eFigure 1 in the Supplement). Thresholds of pack-years of smoking for both OS and PFS were estimated to be 22 (eFigure 2 in the Supplement). On Cox MVA (eTable 2 in the Supplement), smoking for more than 22 pack-years was associated with worse OS (adjusted HR [aHR], 1.57; 95% CI, 1.11-2.22; *P* = .01) and PFS (aHR, 1.38; 95% CI, 1.00-1.89; *P* = .048). On Fine-Gray MVA (eTable 3 in the Supplement), heavy smoking was associated with DF (aHR, 1.71; 95% CI, 1.02-2.88; *P* = .04), but not LRF (aHR, 1.07; 95% CI, 0.61-1.87; *P* = .82). Similar findings were observed in 148 matched pairs for OS (5-year OS, 52.4% vs 79.0%; HR, 1.89; 95% CI, 1.27-2.80; *P* = .002), PFS (5-year PFS, 49.6% vs 69.3%; HR, 1.50; 95% CI, 1.05-2.14; *P* = .03), LRF (5-year LRF, 15.8% vs 14.4%; HR, 1.11; 95% CI, 0.59-2.06; *P* = .75), and DF (5-year DF, 28.0% vs 12.1%; HR, 2.15; 95% CI, 1.19-3.88; *P* = .01; Table and Figure 3). On both Cox and Fine-Gray MVA models, current smoking was not associated with OS, PFS, LRF, or DF (eTable 2 and 3 in the Supplement).

When 10 pack-years of smoking were used as a threshold, there was no association for OS (aHR, 1.23; 95% CI, 0.83-1.81; *P* = .30), PFS (aHR, 1.11; 95% CI, 0.78-1.57; *P* = .56), LRF (aHR, 1.19; 95% CI, 0.64-2.21; *P* = .58), and DF (aHR, 1.45; 95% CI, 0.82-2.56; *P* = .20). Among 350 patients with available HPV data, 252 patients (72.0%) had HPV-associated head and neck cancer. Of patients with HPV-negative tumors, heavy smoking with more than 22 pack-years was not associated with OS (aHR, 1.23; 95% CI, 0.56-2.71; *P* = .60) and PFS (aHR, 1.23; 95% CI, 0.59-2.56; *P* = .58). Similar findings were also observed among HPV-positive tumors (OS: aHR, 1.21; 95% CI, 0.67-2.20; *P* = .53; PFS: aHR, 1.02; 95% CI, 0.61-1.72; *P* = .94). Current smoking during treatment was not associated with OS and PFS among patients with HPV-negative tumors (OS: aHR, 1.19; 95% CI, 0.54-2.65; *P* = .66; PFS: aHR, 1.77; 95% CI, 0.85-3.71; *P* = .13); current smoking during treatment was associated with worse OS and PFS among patients with HPV-positive tumors (OS: aHR, 2.81; 95% CI, 1.26-6.29; *P* = .01; PFS: aHR, 2.51; 95% CI, 1.22-5.14; *P* = .01).

## Discussion

This cohort study is one of the largest single-institution studies investigating the role of pack-years on cancer treatment outcomes among patients with head and neck cancer who underwent definitive-intent chemoradiation. These findings suggest that pack-years have a continuous dose-response association with OS and PFS outcomes. Patients with more than 22 pack-years of smoking were significantly more likely to have worse OS, PFS, and DF outcomes. Current smoking status was independently associated with adverse outcomes, but only among patients with HPV-associated head and neck cancer.

These findings are consistent with a prior multicenter study.^7^ In particular, both studies showed an HR of 1.0 crossed at approximately 20 pack-years of smoking, with a nearly linear association between survival and pack-years of smoking among lighter smokers.^7^ Our findings are also consistent with other institutional studies showing an important role of 20 pack-years as a threshold.^8,23,24^ However, other studies have found 10^5,9,25^ or 30 to 32^7,26^ pack-years as a threshold. Such heterogeneity might be due to challenges in quantifying tobacco exposure^27^ with the pack-years measure. Many individuals who smoke have made multiple quits of various lengths of time and might not have always smoked the same number of cigarettes per day throughout their smoking career. The measure is also subject to considerable retrospective bias. In addition, pack-years does not incorporate the potential complex interaction among the intensity and duration of smoking, smoking status, and the length of time between quitting and diagnosis and treatment.^1^

In our study, it is also notable that greater pack-years of smoking were associated with worse DF, but not LRF, which is inconsistent with other studies suggesting worse LRF outcome.^9,25,26,28^ This discrepancy in locoregional control may be in part due to an automated, readily accessible smoking cessation program at our center for those with substantial smoking history.^12^ Smoking cessation has been shown to improve locoregional control,^2^ and it may explain comparable locoregional control in our study. More research is needed to determine whether smoking cessation after diagnosis impacts LRF specifically and which treatments for smoking cessation are most effective for patients with head and neck cancer.

Similar to our study suggesting the current smoking status as an independent factor associated with survival among HPV-positive tumors, other studies also showed smoking status was a more significant factor than pack-years of smoking.^4,7,9,28,29,30,31,32^ Given the nature of heterogeneous tobacco compounds, the biological impact of current smoking status is complex and multidimensional.^33^ For instance, emerging studies^34,35,36,37^ showed current smokers have immunosuppressive tumor microenvironments with reduced interferon signaling, tumor infiltration of CD8^+^ T cells, immune checkpoint ligands and receptors, natural killer cells, and dendritic cells. Smoking also worsens tumor hypoxia by increasing a carboxyhemoglobin level^38^ and increases *ABCG2* expression inducing resistance to cisplatin.^39^ Additional studies would be needed to elucidate the downstream effect of smoking further.

In recent years, heavy smokers have been shown to be a heterogeneous patient population, with worsening PFS as pack-years of smoking increase and improving PFS as the length of time between quitting and treatment increases.^7^ As a result, more objective measures, such as weighted magnetic resonance imaging and imaging for tumor hypoxia during the course of treatments, were investigated to identify candidates for treatment de-escalation.^40,41^ Another method to improve patient selection for survival outcomes is to incorporate machine learning to account for complex interactions with other host factors.^42^ Although a phase 3 trial failed to show improved locoregional control with radiation dose escalation,^43^ a recent study also investigated the feasibility of adaptive dose escalation among those with poor survival outcomes.^44^ Further studies would be warranted to improve risk stratification and tailor effective treatment options.

### Limitations

Limitations include the retrospective nature of our study. In our cohort study, only patients who underwent definitive-intent chemoradiation were included, and those who could not quit smoking before chemoradiation were excluded for analysis since they often underwent induction chemotherapy at our institution. Our findings may not be generalizable to other patients with head and neck cancer who continued to smoke cigarettes during radiation therapy or underwent surgery, induction chemotherapy, or radiation therapy alone. In addition, patients who quit smoking often relapse,^45^ and misreporting is common when biochemical verification is performed among patients who reported quitting smoking.^46,47^ Self-reported smoking history may not be reliable in select patients. Despite this limitation, many ongoing clinical trials (eg, NRG HN005^48^) on de-escalating treatments among p16-positive oropharyngeal cancer include an eligibility criterion of less than 10 pack-years of tobacco smoking, which is also provided by patients. To our knowledge, there is no objective, validated tool to confirm or deny pack-years of smoking reported by patients that has been incorporated in cooperative group phase 3 trials, and the extent of misreporting may be just as common among patients enrolled in clinical trials. Although pack-years of smoking are being used more routinely to measure the extent of tobacco smoking, some literature report that both duration and pack-years should be considered.^49^ Because the individual importance of duration and pack-years remained unclear with respect to each other, we decided to incorporate the pack-years of smoking only in our study similar to NRG HN002^50^ and NRG HN005^48^ trials.

In addition, the impact of smoking status among HPV-negative tumors is challenging to investigate due to a small sample size of nonsmokers with HPV-negative tumors as shown in our study (9 out of 98 patients, 9.2%).^25,28,29,30^ Small subgroup sample sizes may also explain a lack of significance in 22 pack-years of smoking as a threshold when evaluated separately according to HPV status. Fairly small sample sizes of 134 never smokers (25.9%) and 97 current smokers (18.7%) in our cohort may also explain the wide 95% CIs observed in our results. Despite the reduction of selection bias using propensity score matching, matched patients had smaller sample sizes with reduced statistical power. Furthermore, although alcohol intake has been shown to be associated with local failure and survival outcomes,^3^ the extent of alcohol intake was not included in our database. Our results may not be also generalizable to low-resource facilities without active smoking cessation programs, since nearly half of patients previously reported no smoking cessation counseling^51^ and the efficacy of treatments may vary according to the extent of smoking.^1,2,3,4^

## Conclusions

In our single-institution study, heavy smoking with more than 22 pack-years was an independent, adverse factor for survival and distant metastasis outcomes. Among those with HPV-associated head and neck cancer, current smoking status was also an adverse factor for survival outcomes. Further studies would be warranted to optimize patient selection and tailor treatment strategies.

## Notes

Supplement 1. **eFigure 1. **Nonlinear Cox Regression Model to Evaluate Overall and Progression-Free Survival Outcomes According to Pack-Years of Smoking as a Continuous Variable **eFigure 2.** Distribution of Pack-Years of Smoking and Threshold Evaluation Using Maximum Log-Rank Test Statistic **eTable 1. **Cox Univariable Analysis for Overall and Progression-Free Survival Outcomes According to Human Papillomavirus Status and Smoking History **eTable 2**. Cox Multivariable Analysis for Overall and Progression-Free Survival Outcomes **eTable 3**. Fine-Gray Multivariable Analysis for Locoregional and Distant Failure Outcomes

Supplement 2.
Data Sharing Statement
