# Supplementary material for: Association of Pack-Years of Cigarette Smoking With Survival and Tumor Progression Among Patients Treated With Chemoradiation for Head and Neck Cancer
Source: JAMA Netw Open. 2022 Dec 8;5(12):e2245818. doi: 10.1001/jamanetworkopen.2022.45818 (PMC9856262; doi:10.1001/jamanetworkopen.2022.45818)
Supplement: Supplement 1. — eFigure 1. Nonlinear Cox Regression Model to Evaluate Overall and Progression-Free Survival Outcomes According to Pack-Years of Smoking as a Continuous Variable eFigure 2. Distribution of Pack-Years of Smoking and Threshold Evaluation Using Maximum Log-Rank Test Statistic eTable 1. Cox Univariable Analysis for Overall and Progression-Free Survival Outcomes According to Human Papillomavirus Status and Smoking History eTable 2. Cox Multivariable Analysis for Overall and Progression-Free Survival Outcomes eTable 3. Fine-Gray Multivariable Analysis for Locoregional and Distant Failure Outcomes [file jamanetwopen-e2245818-s001.pdf]

Supplemental Online Content

Ma SJ, Yu H, Yu B, et al. Association of pack-years of cigarette smoking with survival and tumor progression among patients treated with chemoradiation for head and neck cancer. *JAMA Netw Open*. 2022;5(12):e2245818.
doi:10.1001/jamanetworkopen.2022.45818

eFigure 1. Nonlinear Cox Regression Model to Evaluate Overall and Progression-Free Survival Outcomes According to Pack-Years of Smoking as a Continuous Variable
eFigure 2. Distribution of Pack-Years of Smoking and Threshold Evaluation Using Maximum Log-Rank Test Statistic
eTable 1. Cox Univariable Analysis for Overall and Progression-Free Survival Outcomes According to Human Papillomavirus Status and Smoking History
eTable 2. Cox Multivariable Analysis for Overall and Progression-Free Survival Outcomes
eTable 3. Fine-Gray Multivariable Analysis for Locoregional and Distant Failure Outcomes

This supplemental material has been provided by the authors to give readers additional information about their work.

© 2022 MA SJ et al. *JAMA Network Open.*

eFigure 1. Nonlinear Cox Regression Model to Evaluate Overall and Progression-Free Survival Outcomes According to Pack-Years of Smoking as a Continuous Variable

A: progression-free survival; B: overall survival; shaded areas represent 95% confidence interval

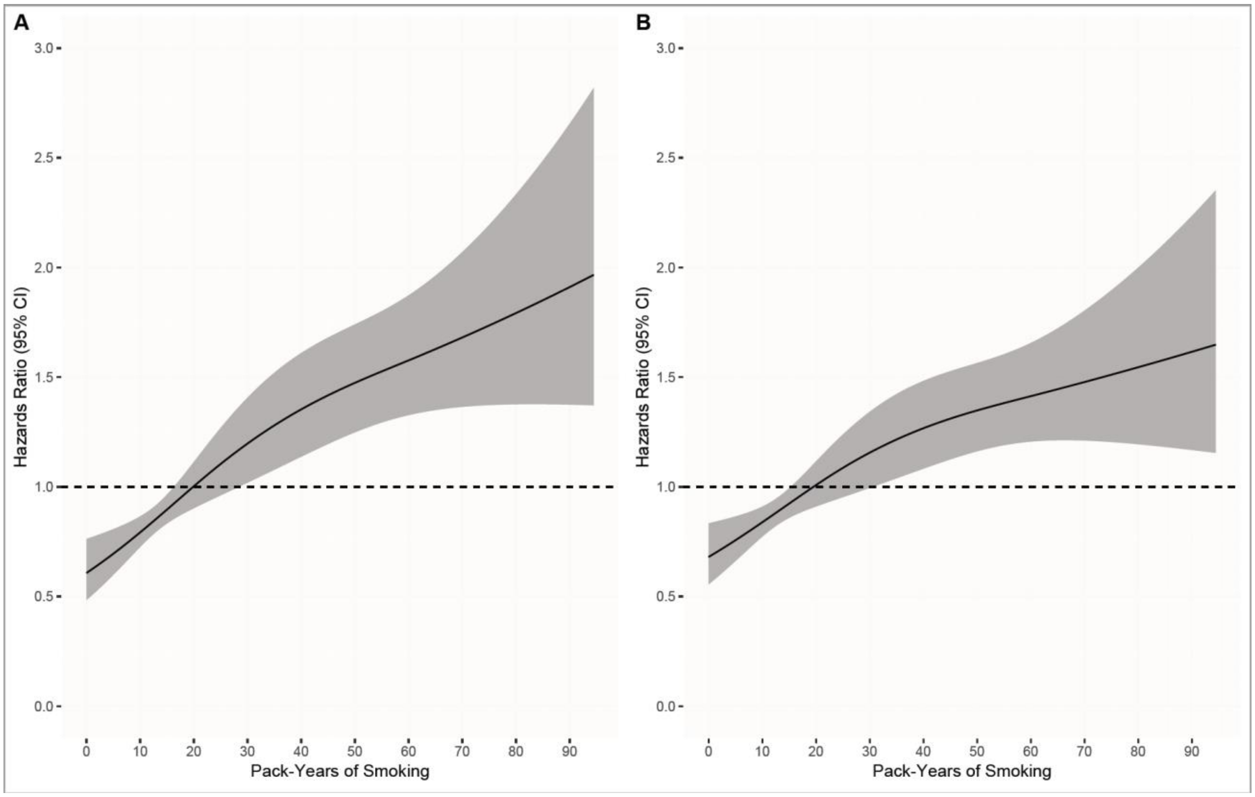

© 2022 MA SJ et al. *JAMA Network Open.*

eFigure 2. Distribution of Pack-Years of Smoking and Threshold Evaluation Using Maximum Log-Rank Test Statistic

OS: overall survival; PFS: progression-free survival

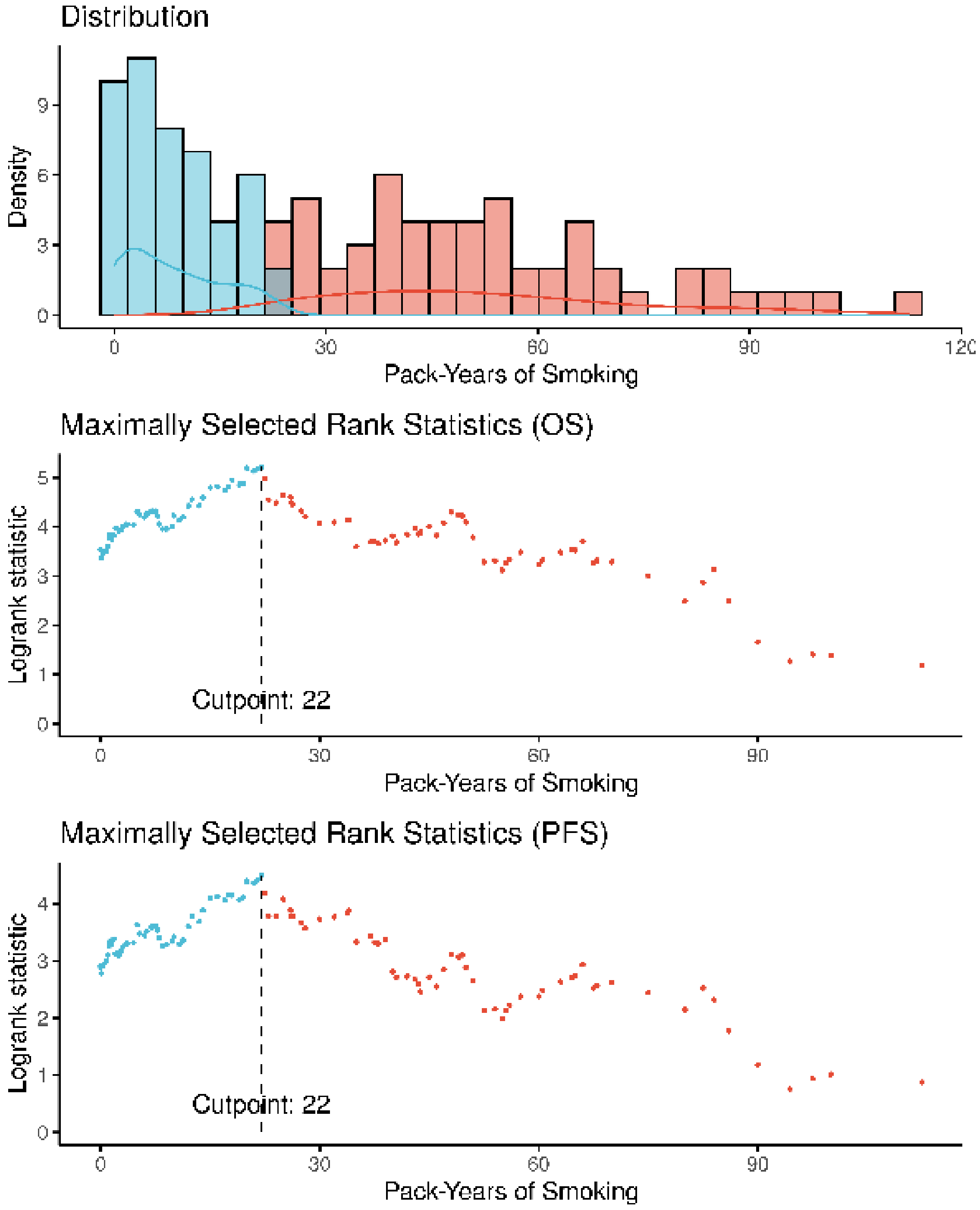

© 2022 MA SJ et al. *JAMA Network Open.*

eTable 1. Cox Univariable Analysis for Overall and Progression-Free Survival Outcomes According to Human Papillomavirus Status and Smoking History

HPV Status	Pack-Years of Smoking	Overall Survival			Progression-Free Survival		
		HR	95% CI	P	HR	95% CI	P
Positive	<10	Reference			Reference		
Positive	10 or more	1.55	0.92-2.62	0.10	1.22	0.78-1.93	0.38
Negative	<10	1.22	0.42-3.53	0.72	0.96	0.34-2.73	0.95
Negative	10 or more	2.79	1.67-4.66	<0.001	2.39	1.53-3.72	<0.001
HPV Status	Smoking Status						
Positive	Never/former	Reference			Reference		
Positive	Current	2.36	1.22-4.54	0.01	1.84	0.99-3.40	0.05
Negative	Never/former	2.23	1.43-3.47	<0.001	1.95	1.30-2.94	0.001
Negative	Current	2.12	1.13-4.00	0.02	2.40	1.38-4.15	0.002

HPV: human papillomavirus; HR: hazards ratio; CI: confidence interval

© 2022 MA SJ et al. *JAMA Network Open.*

eTable 2. Cox Multivariable Analysis for Overall and Progression-Free Survival Outcomes

	Overall survival			Progression-free survival		
	HR	95% CI	P	HR	95% CI	P
Smoking in pack-years						
<22	Reference			Reference		
22 or more	1.57	1.11-2.22	0.01	1.38	1.00-1.89	0.048
Gender						
Male	Reference			Reference		
Female	0.91	0.61-1.37	0.66	0.93	0.64-1.34	0.68
Smoker						
Never or former	Reference			Reference		
Current	1.32	0.89-1.94	0.16	1.27	0.89-1.82	0.19
Age						
For every 1 year older	1.03	1.01-1.04	0.005	1.02	1.00-1.03	0.04
KPS						
<90	Reference			Reference		
90-100	0.69	0.49-0.96	0.03	0.76	0.55-1.03	0.08
Race						
White	Reference			Reference		
Other	1.74	1.15-2.62	0.009	1.47	1.00-2.17	0.05
Comorbidity						
0	Reference			Reference		
1-3	0.54	0.34-0.85	0.008	0.56	0.37-0.84	0.005
4+	0.93	0.56-1.54	0.77	0.84	0.53-1.34	0.47
Site						
Oropharynx	Reference			Reference		
Larynx	0.9	0.57-1.41	0.64	0.91	0.60-1.38	0.64
Other	1.16	0.77-1.76	0.48	1.14	0.78-1.67	0.49
T staging						
1-2	Reference			Reference		

© 2022 MA SJ et al. *JAMA Network Open.*

	Overall survival			Progression-free survival		
	HR	95% CI	P	HR	95% CI	P
3-4	2.23	1.59-3.12	<0.001	1.96	1.45-2.64	<0.001
N staging						
0-1	Reference			Reference		
2-3	1.76	1.18-2.63	0.006	1.86	1.29-2.69	<0.001
HPV						
Negative	Reference			Reference		
Positive	0.64	0.40-1.03	0.06	0.58	0.38-0.88	0.01
Chemo						
Cisplatin	Reference			Reference		
Other	1.54	0.99-2.39	0.05	1.74	1.17-2.58	0.006

HR: hazards ratio; CI: confidence interval; KPS: Karnofsky performance status; HPV: human papillomavirus

© 2022 MA SJ et al. *JAMA Network Open.*

eTable 3. Fine-Gray Multivariable Analysis for Locoregional and Distant Failure Outcomes

	Locoregional failure			Distant failure		
	HR	95% CI	P	HR	95% CI	P
Smoking in pack-years						
<22	Reference			Reference		
22 or more	1.07	0.61-1.87	0.82	1.71	1.02-2.88	0.04
Gender						
Male	Reference			Reference		
Female	0.91	0.46-1.81	0.79	0.59	0.29-1.23	0.16
Smoker						
Never or former	Reference			Reference		
Current	1.01	0.54-1.92	0.97	1.09	0.60-1.98	0.78
Age						
For every 1 year older	1	0.98-1.03	0.82	1	0.97-1.02	0.7
KPS						
<90	Reference			Reference		
90-100	0.8	0.44-1.44	0.46	0.69	0.40-1.19	0.18
Race						
White	Reference			Reference		
Other	2.05	1.04-4.06	0.04	1.35	0.71-2.57	0.35
Comorbidity						
0	Reference			Reference		
1-3	0.82	0.38-1.75	0.61	0.83	0.42-1.66	0.6
4+	0.47	0.17-1.27	0.14	1.01	0.45-2.28	0.97
Site						
Oropharynx	Reference			Reference		
Larynx	1.2	0.49-2.97	0.69	1.18	0.61-2.26	0.63
Other	1.67	0.76-3.67	0.2	1.4	0.79-2.49	0.25
T staging						
1-2	Reference			Reference		

© 2022 MA SJ et al. *JAMA Network Open.*

	Locoregional failure			Distant failure		
	HR	95% CI	P	HR	95% CI	P
3-4	2.35	1.32-4.17	0.004	2.12	1.28-3.53	0.004
N staging						
0-1	Reference			Reference		
2-3	1.01	0.54-1.92	0.97	4.06	2.00-8.23	<0.001
HPV						
Negative	Reference			Reference		
Positive	0.57	0.23-1.40	0.22	1.34	0.66-2.70	0.42
Chemo						
Cisplatin	Reference			Reference		
Other	1.25	0.57-2.71	0.58	1.97	1.05-3.71	0.04

HR: hazards ratio; CI: confidence interval; KPS: Karnofsky performance status; HPV: human papillomavirus

© 2022 MA SJ et al. *JAMA Network Open.*
