# Supplementary material for: Association of Pack-Years of Cigarette Smoking With Survival and Tumor Progression Among Patients Treated With Chemoradiation for Head and Neck Cancer
Source: JAMA Netw Open. 2022 Dec 8;5(12):e2245818. doi: 10.1001/jamanetworkopen.2022.45818 (PMC9856262; doi:10.1001/jamanetworkopen.2022.45818)
Supplement: Supplement 2. — Data Sharing Statement [file jamanetwopen-e2245818-s002.pdf]

Data Sharing Statement

Jun Ma. Association of Pack-Years of Cigarette Smoking With Survival and Tumor Progression Among Patients Treated With Chemoradiation for Head and Neck Cancer. *JAMA Netw Open*. Published December 08, 2022. doi:10.1001/jamanetworkopen.2022.45818

Data
Data available: No

Additional Information
Explanation for why data not available: Research data are stored in an institutional repository and will be shared upon request to the corresponding author.
